# Association of HOTAIR Polymorphisms with Susceptibility to Psoriasis in a Chinese Han Population

**DOI:** 10.1155/2021/5522075

**Published:** 2021-08-04

**Authors:** Xinyu Yao, Siyu Hao, Tiankuo Xue, Keren Zhou, Yu Zhang, Hang Li

**Affiliations:** ^1^Department of Dermatology, Peking University First Hospital, Beijing, China; ^2^Department of Dermatology, The Second Affiliated Hospital of Harbin Medical University, Harbin, Heilongjiang, China

## Abstract

Psoriasis is a common disease in dermatology, but its etiology and pathogenesis have not been fully elucidated. In recent years, researchers have found that HOX transcript antisense RNA (HOTAIR) plays an important role in biological processes as an important long-chain noncoding RNA (lncRNA). The goal of this study was to investigate the association between HOTAIR polymorphisms and psoriasis in a Chinese Han population by screening key candidate single-nucleotide polymorphism (SNPs) sites in HOTAIR. A total of 269 patients diagnosed with psoriasis and 273 healthy control subjects were enrolled in this case-control study. Three SNPs of HOTAIR were genotyped: SNP1 (rs12826786), SNP2 (rs1899663), and SNP3 (rs4759314). All polymorphisms were in Hardy-Weinberg equilibrium in both the control and patient groups, and the SNPs were in linkage disequilibrium. The distribution of the rs4759314 genotype in the control group and case group was statistically significant according to all the models except the recessive model (adjusted *p* value < 0.05), and the CCG haplotype group had a significant difference (OR (95%CI) = 2.907 (1.344 − 6.289), adjusted *p* value = 0.0263). rs12826786 was associated with a risk of psoriasis according to the dominant model (C/T-T/T vs. C/C: OR (95%CI) = 0.70 (0.48 − 1.01), adjusted *p* value = 0.049) and overdominant model (C/T vs. C/C-T/T: OR (95%CI) = 0.69 (0.47 − 1.01), adjusted *p* value = 0.048). The current work showed that a genomic variant within HOTAIR was associated with a risk of psoriasis, and the clinical value of this study should be further evaluated in the future.

## 1. Introduction

Psoriasis is a chronic, recurrent, inflammatory, and systemic disease induced by genetic and environmental factors. The typical clinical manifestation is scaly erythema or plaque, which usually severely affects the quality of life of patients, as there is still a lack of ideal treatment [1]. Psoriasis is a common immune-mediated systemic disease with obvious characteristics of aberrant keratinization of keratinocytes, thick erythematous scale formation, and marked inflammatory cell infiltration. The pathogenesis of psoriasis involves many factors, such as genetic, immune, and environmental abnormalities [2]. Anomalous inflammatory responses and imbalanced inflammatory cytokines induce and advance the pathogenesis of psoriasis [3].

Epigenetics is the discipline that focuses on the genetic changes in gene expression without changes in DNA sequence; the main mechanisms include DNA methylation, histone modification, and noncoding RNA regulation [4]. In recent years, much evidence for epigenetic dysregulation contributing to psoriasis susceptibility and the factors responsible for epigenetic modifications have been summarized [5]. Long noncoding RNAs (lncRNAs), relatively newly discovered noncoding RNAs with a length of more than 200 nucleotides, have no protein coding potential but are a new major regulator of human diseases [6]. The study of epigenetics may provide new ideas and prospects for the treatment of psoriasis.

The HOX transcript antisense RNA (HOTAIR) gene, located on chromosome 12, acts as a scaffold to assemble regulators of the HOXD gene cluster by binding the RNA products of lysine-specific demethylase 1 (LSD1) and polyclonal inhibition complex 2 (PRC2), thus promoting epigenetic inhibition of HOXD [7]. HOTAIR is a long noncoding RNA that is closely related to tumorigenesis and participates in the management of cell proliferation and apoptosis, tumor cell metabolism and metastasis, angiogenesis, and DNA repair [8]. HOTAIR has various functions in the formation of pathological process, especially in the tumorigenesis process [9]. The expression of HOTAIR is frequently upregulated in different kinds of human cancer, including esophageal carcinoma, breast cancer, and lung and gastrointestinal tract carcinomas. HOTAIR has been shown to be an important predictor of poor prognosis for a variety of cancers, including multiple tumors: melanoma, head and neck cancers, urinary tract cancers, digestive system cancers, some female cancers (such as carcinoma of uterine cervix, carcinoma of ovary and endometrial cancers), and hematologic and lymphoid neoplasms [10]. Besides these, previous studies have shown that HOTAIR also affects the progression of nonneoplastic diseases by regulating the expression of inflammatory genes and several cytokines, such as tumor necrosis factor *α* (TNF*α*), IL-6 (interleukin-6), macrophage inflammatory protein-1B (MIP-1B), and inducible nitric oxide synthase (iNOS) [11].

Single-nucleotide polymorphisms (SNPs) are defined as DNA sequence polymorphisms rooted in single-nucleotide variations at the genomic level [12]. HOTAIR has various SNP sites, such as rs12826786, rs1899663, and rs4759314, that possibly affect the expression and function of this lncRNA. Several single-nucleotide polymorphisms analyses have indicated that multiple loci of HOTAIR are closely related to the pathogenesis of disease. Previous studies have pointed out the genotype-specific effect of the rs12826786 SNP on HOTAIR expression in gastric cancer [13] and the association between the rs4759314 minor allele and high HOTAIR transcription in prostate cancers [14]. Correlations of rs1899663 and rs12826786 with prostate diseases [15] and the association between rs12826786 and the risk of autism spectrum disorder have been reported [16]. However, the relationship between HOTAIR and psoriasis still lacks sufficient evidence. Here, we conducted a comprehensive study to investigate and analyze the association between HOTAIR expression and psoriasis in Chinese psoriasis patients.

## 2. Materials and Methods

### 2.1. Patients and Controls

The current case-control study was implemented on samples stemming from 269 patients with psoriasis (104 females, 165 males, age (mean ± SD) = 38.15 ± 9.91) and 273 healthy subjects (125 females, 175 males, age (mean ± SD) = 36.98 ± 10.0). All the samples were obtained from the Second Affiliated Hospital of Harbin Medical University and diagnosed through histopathological examination of paraffin-embedded tissue samples. Genotyping was conducted on blood samples from control subjects and tissue samples of patients. Basic information of all subjects, including gender, age, and demographic characteristic, including age of onset, severity for Psoriasis Area and Severity Index (PASI) scores, and family history (including third-degree relatives), was collected from self-reported questionnaires and medical records (Table S1). Subjects with systemic, infectious, autoimmune, atopic, or malignant diseases were excluded. We calculated the sample size to confirm the reliability of the experimentally data results. All the patients included in this study had a diagnostic criteria for psoriasis vulgaris (PsV) as determined by two professional dermatologists in our institution. The study protocol was authorized by the Ethics Committee of Harbin Medical University, and informed consent forms were signed by all the study participants.

### 2.2. Genetic Studies

We used the international haploid project database (http://hapmap.ncbi.nlm.nih.gov/) and NCBI single-nucleotide polymorphism database (https://www.ncbi.nlm.nih.gov/projects/SNP/) to select potential functional single-nucleotide polymorphisms. Finally, we selected three representative SNPs, rs12826786, rs1899663, and rs4759314 SNPs, for which the Minor Allele Frequency (MAF) was greater than 0.05 in the HapMap Chinese Han Beijing population. We used genomic DNA to extract samples from paraffin-embedded tissue blocks of the disease group and peripheral blood samples of the healthy control group. Genomic DNA was collected from 2 mL of intravenous whole blood using the QIAamp DNA Blood Mini Kit (Qiagen, Hilden, Germany) according to the manufacturer's protocols. DNA samples were followed stored in a freezer (Mitsubishi, Japan) at −20°C. The rs12826786, rs1899663, and rs4759314 SNPs in all the participants were genotyped for our study by tetraprimer amplification refractory mutation system PCR. Taq 2x red master mix was applied for pretreatment, and we performed cycling reactions via a FlexCycler system. All the genotyping experiments were carried out using the improved multiplex ligation reaction (iMLDR) method as described by Genesky Biotechnologies Inc. (Shanghai, China).

### 2.3. Statistical Methods

The genotype and allele frequencies were calculated by direct counts. Deviation from the Hardy-Weinberg equilibrium (HWE) was calculated using chi-square tests. Similarly, Pearson's chi-square test was utilized to compare genotype and allele frequencies between the psoriasis patients and the control people using SPSS 20.0 (SPSS, Inc., Chicago, IL, USA). Odds ratios (ORs) and 95% confidence intervals (CIs) were also calculated, and the associated analyses were performed. Haplotype frequencies for HOTAIR were calculated using SNPStats [17] (http://bioinfo. http://iconcologia.net/SNPstats) based on the expectation maximization algorithm. Pairwise linkage disequilibrium (LD) was assessed by calculating the *D*′ and squared correlation (r2) via SHEsis [18] (http://shesisplus.bio-x.cn/SHEsis.html). *D*′ was determined as the ratio of the unstandardized *D* to its maximal/minimal value. *p* values were rectified by the Bonferroni correction test. When *p* was <0.05, the difference was considered significant.

## 3. Results

Genotype frequencies were in accordance with the HWE supposition in both the patient group and the control group for the three SNPs: rs12826786, rs1899663, and rs4759314 (Table 1). rs12826786 was associated with the risk of psoriasis according to the dominant model (C/T-T/T vs. C/C: OR (95%CI) = 0.70 (0.48 − 1.01), adjusted *p* value = 0.049) and overdominant model (C/T vs. C/C-T/T: OR (95%CI) = 0.69 (0.47 − 1.01), adjusted *p* value = 0.048). The distribution of the rs4759314 genotype in the control and case groups was not statistically significant according to the recessive model but increased the risk of psoriasis according to the other models: codominant model (adjusted *p* value = 0.0021), dominant model (adjusted *p* value = 0.0021), and overdominant model (adjusted *p* value = 0.0009). rs1899663 was not associated with the risk of psoriasis according to any inheritance model (Table 2). The rs12826786, rs1899663, and rs4759314 SNPs were not in LD in the investigated samples (Table 3). Compared with the distribution characteristics of the four haplotypes in the control group and the case group, the CCG haplotype group was significantly different (OR (95%CI) = 2.907 (1.344 − 6.289), adjusted *p* value = 0.0263) (Table 4). We calculated the power of the genotype distribution using the software PASS11 and found that the power of the genotype distribution of the rs4759314 SNP was 0.8829 and that of the rs12826786 SNP in the dominant model was 0.4952 and in the overdominant model was 0.4778.

## 4. Discussion

A previous study suggested that rs12826786 was interrelated with the risk of psoriasis in Iran according to a dominant model (TC + TT vs. CC: OR (95%CI) = 1.59 (0.1.14–2.22), adjusted *p* value = .02) [11]. The T allele of this SNP significantly increased the danger compared with that of the C allele of Iranian psoriasis patients according to the allelic model (OR (95%CI) = 1.35 (1.06–1.71), adjusted *p* value = .04) [11]. However, there is a lack of thorough and effective studies that have explored correlations between SNPs and the risk of developing psoriasis. In this study, we genotyped three SNPs for HOTAIR in 269 psoriasis patients and 273 healthy controls, detected the correlation of rs12826786, rs4759314, and rs1899663 with the disease, and analyzed the risk of psoriasis according to different inheritance models. There was a significant difference in allele frequency for rs4759314 and after that we made different genetic models of dominant, recessive, codominant, and overdominant, which also suggested that this locus was associated with psoriasis. In addition, we first found that the CCG haplotype might decrease the risk of psoriasis in our results. On the other hand, there was no difference in allele frequency for 12826786 between patients and normal subjects, but codominant model and dominant model suggested that this locus was also associated with psoriasis. We first found that the SNP at the HOTAIR locus was associated with a risk of psoriasis in a selected Chinese Han population, which might provide a new potential target for the treatment of psoriasis. It is indicated that the samples selected in this paper could represent the population level of the Chinese Han population to a certain extent.

We speculate that these HOTAIR SNPs might affect the susceptibility of psoriasis by changing the level of inflammatory factors, which may be explained by the role of and the subsequent imbalance of the cytokine network and inflammatory factors attributed to the development of the pathological process of psoriasis. HOTAIR is a major regulator of multiple biological process and appears to play critical roles in the cell signaling transduction and immune response. The function of regulation for a variety of inflammatory molecules such as TNF*α*, IL-6, MIP-1B, and iNOS has been reported, which also plays an important role in the pathogenesis of psoriasis [11]. The increased expression and production of IL-6 leads to an increase in interleukin-17A in the skin, which in turn leads to the aggregation of neutrophils and other granulo-monocytes at the inflammatory site [19]. NOS may be essential in the process of skin tissue repair and regulation of skin cell proliferation. In psoriatic skin, compared to normal skin, nNOS and iNOS were diffusely overexpressed, while eNOS was strongly stained in endothelial cells [20]. TNF-*α* has a crucial role in the development and maintenance of psoriatic plaques, and its serum levels correlate with disease activity. Biologic therapies involving TNF-*α* antagonists are widely used for the long-term management of moderate to severe vulgaris psoriasis in clinical practice [21]. It was also found that the overexpression of HOTAIR promoted cell injury and activated PI3K/AKT and NF-*κ*B pathways by upregulation of double-stranded RNA-dependent protein kinase (PKR) in UVB-injured HaCaT cells [22]. These changes in the expression of inflammatory factors may lead to abnormal changing of keratinocytes finally resulting to the occurrence and development of psoriasis.

Overall, our study is a novel and progressive approach to further determining the association between the genomic variant of HOTAIR and risk of psoriasis incidence. Moreover, this is the first clinical study on psoriasis of the Chinese Han nationality. The results of this study contribute to the discovery of diagnostic and therapeutic targets for this disease, but it is necessary to further evaluate the expression level of biomarkers in blood samples and skin tissues of psoriasis patients and to explore the related mechanisms in vivo and in vitro. A prospective study with a larger sample size than that used in the present study is needed to further verify our findings.

## Figures and Tables

**Table 1:** Results of Hardy-Weinberg equilibrium.

		*p*
rs12826786	Cases	0.340
	Controls	0.850
	Totals	0.760
rs1899663	Cases	0.810
	Controls	1.000
	Totals	1.000
rs4759314	Cases	1.000
	Controls	0.065
	Totals	0.410

*p*: model-based statistical *p* value.

**Table 2:** Results of association analysis between HOTAIR SNP genotypes and psoriasis.

			Cases	Controls	OR (95% CI)	*p*	*p* ^∗^	OR (95% CI)^#^	*p* ^#^
rs12826786	Codominant	C/C	178 (65.2%)	196 (72.9%)	1.0	0.150	0.600	1.0	0.13
		C/T	86 (31.5%)	65 (24.2%)	0.69 (0.47-1.00)			0.68 (0.46-0.99)	
		T/T	9 (3.3%)	8 (3%)	0.81 (0.30-2.14)			0.84 (0.31-2.23)	
	Allele	C	442 (81.0%)	457 (84.9%)	1.0	0.081	0.324		
		T	104 (19.0%)	8 1 (15.1%)	0.7 5 (0.55-1.04)				
	Dominant	C/C	178 (65.2%)	196 (72.9%)	1.0	0.054	0.216	1.0	0.049
		C/T-T/T	95 (34.8%)	73 (27.1%)	0.70 (0.48-1.01)			0.69 (0.48-1.00)	
	Recessive	C/C-C/T	264 (96.7%)	261 (97%)	1.0	0.830	1.000	1.0	0.88
		T/T	9 (3.3%)	8 (3%)	0.90 (0.34-2.37)			0.93 (0.35-2.46)	
	Overdominant	C/C-T/T	187 (68.5%)	204 (75.8%)	1.0	0.056	0.224	1.0	0.048
		C/T	86 (31.5%)	65 (24.2%)	0.69 (0.47-1.01)			0.68 (0.47-1.00)	
rs1899663	Codominant	C/C	180 (65.9%)	197 (73.2%)	1.0	0.170	0.680	1.0	0.16
		C/A	84 (30.8%)	66 (24.5%)	0.72 (0.49-1.05)			0.71 (0.48-1.04)	
		A/A	9 (3.3%)	6 (2.2%)	0.61 (0.21-1.75)			0.62 (0.22-1.79)	
		C	444 (81.32%)	460 (85.50%)	1.0	0.065	0.260		
		A	102 (18.68%)	78 (14.50%)	0.74 (0.54-1.02)				
	Dominant	C/C	180 (65.9%)	197 (73.2%)	1.0	0.064	0.256	1.0	0.059
		C/A-A/A	93 (34.1%)	72 (26.8%)	0.71 (0.49-1.02)			0.70 (0.48-1.01)	
	Recessive	C/C-C/A	264 (96.7%)	263 (97.8%)	1.0	0.450	1.000	1.0	0.47
		A/A	9 (3.3%)	6 (2.2%)	0.67 (0.23-1.91)			0.68 (0.24-1.950)	
	Overdominant	C/C-A/A	189 (69.2%)	203 (75.5%)	1.0	0.100	0.400	1.0	0.093
		C/A	84 (30.8%)	66 (24.5%)	0.73 (0.50-1.07)			0.72 (0.49-1.06)	
rs4759314	Codominant	A/A	265 (97.1%)	244 (90.7%)	1.0	0.002	0.008	1.0	0.0021
		G/A	7 (2.6%)	25 (9.3%)	3.88 (1.65-9.13)			3.78 (1.60-8.94)	
		G/G	1 (0.4%)	0 (0%)	—			—	
		A	537 (98.4%)	513 (95.4%)	1.0	0.007	0.028		
		G	9 (1.6%)	25 (4.6%)	2.91 (1.34-6.29)				
	Dominant	A/A	265 (97.1%)	244 (90.7%)	1.0	0.002	0.008	1.0	0.0021
		G/A-G/G	8 (2.9%)	25 (9.3%)	3.39 (1.50-7.67)			3.30 (1.46-7.48)	
	Recessive	A/A-G/A	272 (99.6%)	269 (100%)	1.0	0.240	0.960	1.0	0.25
		G/G	1 (0.4%)	0 (0%)	—			—	
	Overdominant	A/A-G/G	266 (97.4%)	244 (90.7%)	1.0	0.001	0.004	1.0	0.0009
		G/A	7 (2.6%)	25 (9.3%)	3.89 (1.65-9.16)			3.79 (1.60-8.96)	

SNP: single-nucleotide polymorphism, *p*: model-based statistical *p* value, *p*∗: *p* value adjusted Bonferroni correction, *p*^#^: *p* value adjusted by age and sex; OR: odds ratio; 95% CI: 95% confidence interval.

**Table 3:** *D*′ and *r* statistics for the assessment of linkage disequilibrium between rs12826786, rs1899663, and rs4759314 SNPs.

*D*′			
	rs12826786	rs1899663	rs4759314
rs12826786	—	0.993	0.997
rs1899663	—	—	0.997
rs4759314	—	—	—
R^2^			
	rs12826786	rs1899663	rs4759314
rs12826786	—	0.955	0.007
rs1899663	—	—	0.006
rs4759314	—	—	—

**Table 4:** Results of haplotype analysis.

rs12826786	rs1899663	rs4759314	Total	Frequency in control	Frequency in cases	OR (95% CI)	*p*	*p* ^∗^	OR (95% CI)^#^	*p* ^#^
C	C	A	0.797	0.7912	0.803	1.075 (0.800~1.446)	0.631	1.00	1.075 (0.799-1.446)	0.63
C	C	G	0.0314	0.0165	0.0465	2.913 (1.346~6.304)	0.005	0.037	2.907 (1.344-6.289)	0.004
T	A	A	0.1651	0.185	0.145	0.747 (0.541~1.032)	0.076402	0.608	0.747 (0.54-1.031)	0.076
T	C	A	0.0055	0.0055	0.0056	1.013 (0.204~5.037)	0.987	1.00	1.014 (0.203-5.051)	0.985

^∗^: *p* value after Bonferroni correction; ^#^The OR and *p* values were adjusted for age and sex.

## Data Availability

The data used to support the findings of this study are available from the corresponding author upon request.

## Supplementary Materials

Supplementary Materials Table S1: The basic demographic and clinical data for the participants.

## References

[1] Ran D.-L., Cai M., Zhang X. (2019). Genetics of psoriasis: a basis for precision medicine. *Precision Clinical Medicine*.

[2] Yao Y., Richman L., Morehouse C. (2008). Type I interferon: potential therapeutic target for psoriasis?. *PLoS One*.

[3] Sabat R., Philipp S., Höflich C. (2007). Immunopathogenesis of psoriasis. *Experimental Dermatology*.

[4] Calcagno D. Q., Gigek C. O., Chen E. S., Burbano R. R., Smith Mde A. (2013). DNA and histone methylation in gastric carcinogenesis. *World Journal of Gastroenterology*.

[5] Wu H., Chen Y., Zhu H., Zhao M., Lu Q. (2019). The pathogenic role of dysregulated epigenetic modifications in autoimmune diseases. *Frontiers in Immunology*.

[6] Nagano T., Fraser P. (2011). No-nonsense functions for long noncoding RNAs. *Cell*.

[7] Rinn J. L., Kertesz M., Wang J. K. (2007). Functional demarcation of active and silent chromatin domains in human HOX loci by noncoding RNAs. *Cell*.

[8] Yu X., Li Z. (2015). Long non-coding RNA HOTAIR: a novel oncogene (review). *Molecular Medicine Reports*.

[9] Tang Q., Hann S. S. (2018). HOTAIR: an oncogenic long non-coding RNA in human cancer. *Cellular Physiology and Biochemistry*.

[10] Toy H. I., Okmen D., Kontou P. I., Georgakilas A. G., Pavlopoulou A. (2019). HOTAIR as a prognostic predictor for diverse human cancers: a meta- and bioinformatics analysis. *Cancers (Basel)*.

[11] Rakhshan A., Zarrinpour N., Moradi A. (2020). A single nucleotide polymorphism within HOX transcript antisense RNA (HOTAIR) is associated with risk of psoriasis. *International Journal of Immunogenetics*.

[12] Sachidanandam R., Weissman D., Schmidt S. C. (2001). A map of human genome sequence variation containing 1.42 million single nucleotide polymorphisms. *Nature*.

[13] Guo W., Dong Z., Bai Y. (2015). Associations between polymorphisms of HOTAIR and risk of gastric cardia adenocarcinoma in a population of North China. *Tumour Biology*.

[14] Jiang D., Xu L., Ni J., Zhang J., Cai M., Shen L. (2019). Functional polymorphisms in LncRNA HOTAIR contribute to susceptibility of pancreatic cancer. *Cancer Cell International*.

[15] Khorshidi H. R., Taheri M., Noroozi R., Soudyab M., Sayad A., Ghafouri-Fard S. (2017). Investigation of the association of HOTAIR single nucleotide polymorphisms and risk of breast cancer in an Iranian population. *International Journal of Cancer Management*.

[16] Safari M., Noroozi R., Taheri M., Ghafouri-Fard S. (2020). The rs12826786 in HOTAIR lncRNA is associated with risk of autism spectrum disorder. *Journal of Molecular Neuroscience*.

[17] Sole X., Guino E., Valls J., Iniesta R., Moreno V. (2006). SNPStats: a web tool for the analysis of association studies. *Bioinformatics*.

[18] Shi Y. Y., He L. (2005). SHEsis, a powerful software platform for analyses of linkage disequilibrium, haplotype construction, and genetic association at polymorphism loci. *Cell Research*.

[19] Klebow S., Hahn M., Nikoalev A. (2016). IL-6 signaling in myelomonocytic cells is not crucial for the development of IMQ-induced psoriasis. *PLoS One*.

[20] Kim T. G., Kim D. S., Kim H. P., Lee M. G. (2014). The pathophysiological role of dendritic cell subsets in psoriasis. *BMB Reports*.

[21] de Gannes G. C., Ghoreishi M., Pope J. (2007). Psoriasis and pustular dermatitis triggered by TNF-{alpha} inhibitors in patients with rheumatologic conditions. *Archives of Dermatology*.

[22] Liu G., Zhang W. (2018). Long non-coding RNA HOTAIR promotes UVB-induced apoptosis and inflammatory injury by up-regulation of PKR in keratinocytes. *Brazilian Journal of Medical and Biological Research*.

