# Supplementary material for: Association of HOTAIR Polymorphisms with Susceptibility to Psoriasis in a Chinese Han Population
Source: Biomed Res Int. 2021 Aug 4;2021:5522075. doi: 10.1155/2021/5522075 (PMC8357505; doi:10.1155/2021/5522075)
Supplement: Supplementary Materials — Table S1: The basic demographic and clinical data for the participants. [file 5522075.f1.docx]

Table S1: The basic demographic and clinical data for the participants.

| Characteristic | Cases | Controls | p Value |
| --- | --- | --- | --- |
| Total number | 269 | 273 |  |
| Gender, n (%) |  |  | 0.075 |
| Male | 141(0.524) | 151(0.553) |  |
| Female | 128(0.476) | 122(0.447) |  |
| Mean age±SD, years | 42.03±12.13 | 44.26±11.98 | 0.279 |
| PASI, n (%) |  |  |  |
| ≤10 | 484(0.893) |  |  |
| >10 | 58 (0.107) |  |  |
| p Value | 0.109 |  |  |
| Age at onset, n (%) |  |  |  |
| ≤40 year | 429 (0.792) |  |  |
| >40 years | 113 (0.208) |  |  |
| p Value | 0.221 |  |  |
| Family history, n (%) |  |  |  |
| Yes | 203 (0.375) |  |  |
| No | 337 (0.625) |  |  |
| p Value | 0.113 |  |  |
